# Injection of the Constituents of Bile into the Veins of an Animal

**Published:** 1870-04

**Authors:** 


M. Salmon de Champotran, an official of the Council of State (Paris), who died a few months since, has left to the Faculty of Medicine of Paris, a legacy to found a chair of The History of Medicine. The contest for the chair is warm, the most prominent candidate being M. Bouchut, of the children's hospital, so well and favorably known by his clinical lectures, by his investigations in general pathology, and his Treatise on the History of Medicine and Medical Doctrines. — *Gazette des Hopitaux.*

M. See, in his introductory to the course of lectures upon the Application of Physiology to Medicine, at l'Hopital la Charité (Paris), gives the following as the results of the injection of the constituents of bile into the veins of an animal.

By separating the coloring matter and injecting the biliary acids, the following series of constant phenomena are produced:

1st. The retardation of the pulse by the excitation of the vagus nerve; the pneumo-gastric nerve being the antagonist of the motor ganglia of the heart, its excitation induces a retardation (of the pulse), which is likewise produced by the biliary acids exactly as by digitalis, more promptly and more surely, however.

2nd. The cutaneous itching may be explained by the excitation of the extremities of the sensitive nerves of the skin by the cholic and choleic acids.

3rd. The constipation is due to a great extent to the absence of these acids from the intestine. In the normal state they produce upon the muscular plane which is manifested by the contractions of the intestinal muscles; if this excitation is absent, there is retention of the fæces.

The action of the bile itself containing its coloring matter gives other results.

1st. The coloring of the fæcal matters, if this no longer flows into the intestine in consequence of obstruction of the biliary conduits, the stools are discolored, grayish.

2nd. The penetration of the bile into the blood when it finds its natural emunctories obliterated, determines the impregnation of all the tissues, and principally of the very vascular tissues, by biliverdin.

3rd. Once introduced into the blood, it tends to eliminate itself, hence the coloring of the different secreted liquids and more particularly of the urine.

Such is the origin, long misunderstood, of the phenomena which supervene in icteric maladies.

THE same distinguished physiologist explains the syncope (sometimes mortal), resulting from a blow upon the epigastrium, thus:

This violent blow occasions very active excitation of the abdominal segments of the pneumo-gastric nerve, which is transmitted to the medulla to be reflected upon the cardiac filaments of the pneumo-gastric, thereby inducing a diminution or even an arrest of the pulsations of the heart.

Moreover, there is associated with the pneumo-gastric the depressor-cardiac nerve of Cyon. This nerve, when excited, has the power to diminish considerably pressure in the vessels and consquently to weaken the circulation. Now this same excitation originating in the abdomen, involving the pneumo-gastric, is propagated likewise to the nerve of Cyon, and induces, by the intermediation of the medulla oblongata, a paralysis of the splanchnic vaso-motos and a consecutive dilatation of the vascular system; hence simultaneous weakness of the heart and of the vessels; hence diminution of the circulation and consequently cyanosis.

M. ROUSSIN, in a paper read before the Imperial Academy of Medicine, upon hydrate of chloral, its new mode of preparation and the characteristics of its purity, affirms that this agent absorbed or ingested into the economy, in any manner whatever, must be converted rapidly into alkaline formiate or chloroform. — *Gazette des Hopitaux.*

